# A genome-wide screen reveals that Dyrk1A kinase promotes nucleotide excision repair by preventing aberrant overexpression of cyclin D1 and p21

**DOI:** 10.1016/j.jbc.2023.104900

**Published:** 2023-06-09

**Authors:** François Bélanger, Cassandra Roussel, Christina Sawchyn, Edlie St-Hilaire, Sari Gezzar-Dandashi, Aimé Boris Kimenyi Ishimwe, Frédérick Antoine Mallette, Hugo Wurtele, Elliot Drobetsky

**Affiliations:** 1 Centre de Recherche de l’Hôpital Maisonneuve-Rosemont, Montréal, Québec, Canada; 2 Department of Biochemistry and Molecular Medicine, Université de Montréal, Montréal, Québec, Canada; 3 Molecular Biology Program, Université de Montréal, Montréal, Québec, Canada; 4 Department of Medicine, Université de Montréal, Montréal, Québec, Canada

**Keywords:** nucleotide excision repair, Dyrk1A kinase, cyclin D1, p21, malignant melanoma

## Abstract

Nucleotide excision repair (NER) eliminates highly genotoxic solar UV-induced DNA photoproducts that otherwise stimulate malignant melanoma development. Here, a genome-wide loss-of-function screen, coupling CRISPR/Cas9 technology with a flow cytometry-based DNA repair assay, was used to identify novel genes required for efficient NER in primary human fibroblasts. Interestingly, the screen revealed multiple genes encoding proteins, with no previously known involvement in UV damage repair, that significantly modulate NER uniquely during S phase of the cell cycle. Among these, we further characterized Dyrk1A, a dual specificity kinase that phosphorylates the proto-oncoprotein cyclin D1 on threonine 286 (T286), thereby stimulating its timely cytoplasmic relocalization and proteasomal degradation, which is required for proper regulation of the G1-S phase transition and control of cellular proliferation. We demonstrate that in UV-irradiated HeLa cells, depletion of Dyrk1A leading to overexpression of cyclin D1 causes inhibition of NER uniquely during S phase and reduced cell survival. Consistently, expression/nuclear accumulation of nonphosphorylatable cyclin D1 (T286A) in melanoma cells strongly interferes with S phase NER and enhances cytotoxicity post-UV. Moreover, the negative impact of cyclin D1 (T286A) overexpression on repair is independent of cyclin-dependent kinase activity but requires cyclin D1–dependent upregulation of p21 expression. Our data indicate that inhibition of NER during S phase might represent a previously unappreciated noncanonical mechanism by which oncogenic cyclin D1 fosters melanomagenesis.

Nucleotide excision repair (NER) is the preeminent pathway in humans for removing helix-destabilizing, replication- and transcription-blocking DNA adducts generated by a variety of environmental mutagens/carcinogens and chemotherapeutic drugs. Among such adducts are solar UV-induced cyclobutane pyrimidine dimers (CPD) and 6-4 pyrimidine-pyrimidone photoproducts (6-4PP), which represent the overriding cause of mutations promoting cutaneous tumor development (1, 2). NER comprises two overlapping subpathways, differing only in the mechanism of DNA damage recognition. During global genomic NER (GG-NER), helix-destabilizing DNA lesions are recognized throughout the genome by heterotrimeric XPC/HR23B/Centrin2, in collaboration with the DDB2/DDB1/Cul4A ubiquitin E3 ligase complex. The other subpathway, transcription-coupled NER, acting only along the transcribed strand of active genes, is initiated when elongating RNA polymerase II stalls at damaged DNA bases, which in turn promotes recruitment of CSB and the CSA/DDB1/Cul4A ubiquitin E3 ligase complex. After lesion recognition associated with either subpathway, proteins of the “core NER pathway” are sequentially recruited and function as follows: (i) The helicase and ATPase activities of XPD and XPB, respectively, as subunits of the TFIIH basal transcription factor mediate DNA unwinding at the damaged site; (ii) XPA partners with heterotrimeric replication protein A (RPA) to stabilize the unwound DNA and promote lesion verification in collaboration with TFIIH; (iii) The ERCC1-XPF and XPG endonucleases incise the DNA backbone on either side (5′ and 3′, respectively) of the lesion, producing a single-stranded DNA (ssDNA) fragment containing the adduct which is subsequently excised; (iv) the resulting ∼30 bp gap is resynthesized by DNA replication factors using the damage-free complementary strand as template; and, finally, (v) DNA ligases seal the remaining nick to restore the original DNA sequence. (For review of the NER pathway, see (3, 4)).

The syndrome *Xeroderma pigmentosum*, characterized by homozygous germline mutations in NER pathway genes and remarkable (up to 5000-fold increased) susceptibility to sunlight-induced skin cancers including malignant melanoma (MM), underscores the importance of NER to human health (5). In view of this, it may be surprising that multiple genome-wide sequencing studies have revealed a paucity of NER gene defects in sporadic MM within the general population (2, 6, 7)*.* On the other hand, such studies have identified numerous potential MM driver mutations, although in many cases it remains unclear how these mutations promote melanoma development and whether some might do so by negatively impacting NER.

We previously demonstrated that defective responses to DNA replication stress, for example, in cells depleted for ataxia telangiectasia and rad 3-related kinase (ATR) or translesion DNA polymerase eta (pol eta), cause striking GG-NER defects during S phase, whereas repair in G1 or G2/M is not significantly impacted (GG-NER during S phase is hereafter denoted S phase NER) (8, 9, 10, 11). As outlined above, the RPA complex, which binds and protects ssDNA generated during genotoxin-induced replicative stress (12), also plays a critical role in NER (13). Upon severe UV-induced replication stress, excessive sequestration of RPA on ssDNA located at persistently stalled replication forks, or at aberrantly activated origins of replication, was shown by our group and others to limit the availability of this complex to act in NER during S phase (10, 11, 14). This raises the possibility that a multitude of proteins which act to mitigate replicative stress, and hence ssDNA generation, might modulate the efficiency of NER in an S phase–specific manner. Moreover, such proteins may have thus far escaped detection because classical NER assays, for example, quantification of NER gap-filling by unscheduled DNA synthesis (UDS) (15), are not designed to evaluate UV damage repair during S phase.

Here, we employed a flow cytometry-based CRISPR/Cas9 genome-wide screen to identify factors required for efficient removal of UV-induced DNA photoproducts in primary human fibroblasts. Interestingly, this screen revealed multiple proteins that significantly influence NER specifically during S phase, including Dyrk1A kinase which phosphorylates the proto-oncoprotein cyclin D1 on threonine 286 (T286) (16). Failure to modify cyclin D1 on T286 leads to aberrant nuclear accumulation of the protein, premature S phase entry, and enhanced cellular proliferation that drives tumorigenesis (17). We demonstrate here that lack of Dyrk1A inhibits S phase NER and compromises cell survival post-UV by causing overexpression of cyclin D1. Moreover, we show that this does not depend on either modulation of cyclin-dependent kinase (CDK) activity or the induction of DNA replication stress but instead on cyclin D1-dependent upregulation of p21 expression.

## Results

### A CRISPR/Cas9 screen identifies novel genes regulating NER

**Figure 1:**
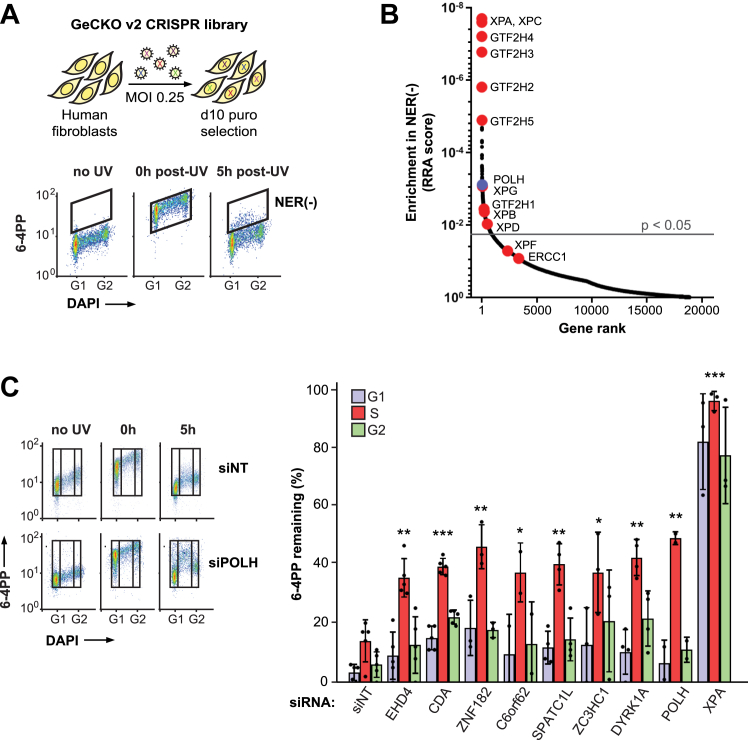
**A CRISPR/Cas9 loss-of-function screen identifies novel NER-regulating genes.***A,* LF-1 primary lung fibroblasts were infected with the GeCKO v2 lentiviral library, irradiated with 20 J/m^2^ of UV, and selected for 10 days in puromycin. A flow cytometry-based NER assay was then employed to sort cells characterized by reduced efficiency of 6-4PP removal (corresponding to the boxed population at 5 h-post UV). *B,* MAGeCK software was used to assign an RRA score and adjusted *p*-value for each gene (see Experimental procedures). Known NER genes are shown in *red* and *POLH* (encoding DNA pol eta) in *blue*. Genes above the *gray line* exhibit *p* values <0.05. *C,* candidate genes were knocked down individually in HeLa cells using siRNA. *Left:* representative bivariate plots of 6-4PP removal as a function of cell cycle for cells treated with siRNA against *POLH* or nontargeting (siNT) control. *Right:* 6-4PP remaining 5 h post-UV as a function of cell cycle for individual candidate genes, as well as for *XPA* and *POLH* (controls), following siRNA knockdown. *C*, data are reported as the mean ± SD for at least three independent experiments. *p*-values compare the percentage of damage remaining in S phase for each siRNA *versus* siNT control, and were obtained using the two-tailed unpaired Student *t* test, adjusted for multiple tests by the Holm-Sidak method; ∗*p* < 0.05, ∗∗*p* < 0.01, ∗∗∗*p* < 0.001. 6-4PP, 6-4 pyrimidine-pyrimidone photoproduct; NER, nucleotide excision repair.

We previously developed a flow cytometry–based immunoassay to directly quantify the repair of 6-4PP as a function of cell cycle (9). Here, this assay was exploited in conjunction with CRISPR/Cas9 technology to perform a genome-wide loss-of-function screen as a means of identifying genes that promote efficient NER. LF-1 primary lung fibroblasts (18) were transduced at low multiplicity of infection with pool A of the GeCKO v2 single-vector system (19), followed by 10 days of puromycin selection to remove uninfected cells. 10^8^ selected cells were then irradiated with 20 J/m^2^ UV, and ones presenting high residual 6-4PP at 5 h post-UV were sorted by flow cytometry (Fig. 1*A*). Although the harsh conditions of our labeling protocol adversely affected the yield of intact single cells, we successfully sorted 1.2 × 10^7^ individual cells resulting in a coverage of approximately 200 genome equivalents. A sample of 30 million unsorted cells was used as control to assess the single-guide RNA (sgRNA) content of the transduced cell population.

sgRNAs were PCR-amplified from control and NER-deficient populations and sequenced. The data were analyzed using MAGeCK software (https://sourceforge.net/projects/mageck/) (20) to identify sgRNAs significantly enriched among NER-defective cells (Fig. 1*B* and Table S1). The top six hits were direct participants in the NER pathway, that is, genes encoding XPA, XPC, and four TFIIH core subunits (GTF2H2-5); moreover, several other NER pathway genes, including *XPB, XPD, XPG,* and *GTF2H1*, exhibited significant *p*-values (<0.05). Importantly, consistent with our previous work showing that functional pol eta is required for efficient S phase NER (8), sgRNAs targeting *POLH* (encoding pol eta) were significantly enriched in the NER-defective fraction. Gene Ontology (GO-term) analysis was performed on the top-ranked 150 genes. As expected, terms associated with NER, DNA repair, and the DNA damage response displayed significant enrichment (Table S2). Moreover, all of the significant non-NER/non-DNA repair GO-terms were populated with NER genes, for example, the GO-term “DNA templated transcription initiation” includes genes encoding TFIIH subunits. Overall, the data show that our screening strategy is competent in identifying *bona fide* NER-modulating genes.

Among the top 50 genes, we selected seven, with no previously known involvement in NER, for validation using HeLa cells as model system. The chosen candidates displayed at least 50% enrichment for all three sgRNAs in the library, in the NER-defective fraction compared to the reference sample: (i) *SPATC1L* (spermatogenesis and centriole-associated protein 1 like), a germ-cell–specific factor implicated in spermiogenesis (21), (ii) *CDA* (cytidine deaminase), involved in the pyrimidine salvage pathway (22), (iii) *DYRK1A* (dual-specificity tyrosine phosphorylation-regulated kinase 1A) (23), (iv) *ZC3HC1* (also known as NIPA; nuclear interaction partner of ALK), a component of an SCF-type E3 ubiquitin ligase complex regulating the G2-M transition (24), (v) *EHD4* (EH domain containing 4), involved in endosomal transport (25), and (vi) *C6orf62* and *ZNF182*, with no established functions. Unexpectedly, siRNA-mediated depletion of each of the above factors engendered significant defects in 6-4PP removal during S phase, but not in G1 or G2, unlike the situation for the NER pathway protein XPA, where repair was defective throughout the cell cycle (Fig. 1*C*; see Table S3 for knockdown efficiencies). We note that, as assessed by 4′,6-diamidino-2-phenylindole (DAPI) staining, the DNA content of S phase HeLa cells did not noticeably increase for at least 5 h post-UV (Fig. S1, *A*–*D*; the nucleoside analog EdU was used to label S-phase cells). Moreover, cells in G1 (EdU-negative) did not enter S phase during the same period. This likely reflects DNA damage–induced inhibition of the G1-S transition and delayed progression through S phase and confirms that our experimental conditions permit accurate quantification of 6-4PP removal during S phase, that is, without any confounding influence of cells irradiated in G1, which might have then progressed to S phase. Overall, the data indicate that the human genome encodes previously unknown NER regulators that promote UV DNA photoproduct removal in an S phase–specific manner.

### Dyrk1A promotes NER specifically during S phase and cell survival post-UV

**Figure 2:**
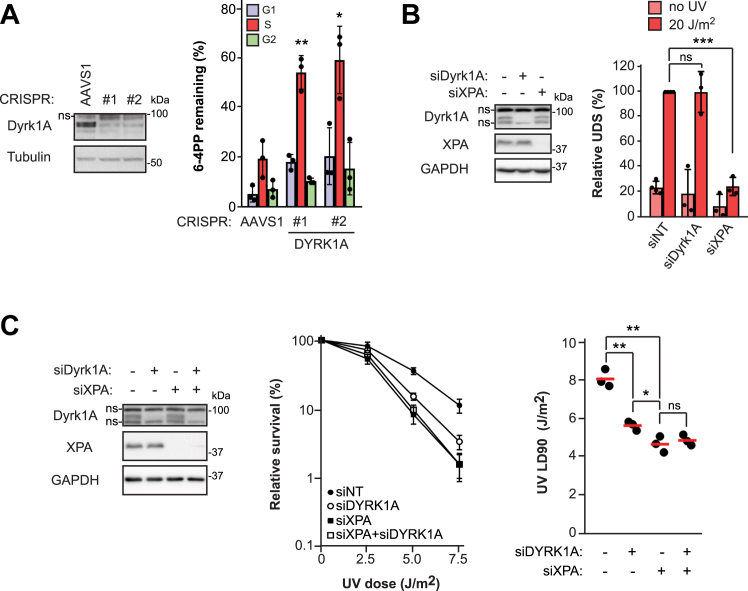
**Influence of Dyrk1A on NER and cell survival post-UV.***A*, *left:* knockout of *Dyrk1A* by CRISPR/Cas9 in LF-1 primary lung fibroblasts. sgRNA against the adeno-associated virus integration site (AAVS1) was used as a negative control. On this and all subsequent immunoblots, “ns” indicates a nonspecific band; *Right:* quantification of 6-4PP removal as in Figure 1*C*. *B*, evaluation of unscheduled DNA synthesis (UDS) post-UV in HeLa cells treated with siRNA against Dyrk1A (siDyrk1A) or XPA (siXPA). *Left*: immunoblots showing knockdown of Dyrk1A and XPA. *Right*: quantification of EdU incorporation in G1/G2 after 20 J/m^2^ UV or mock treatment. *C*, clonogenic survival post-UV in HeLa cells treated with siDyrk1A and/or siXPA. *Left*: immunoblot showing protein knockdown. *Middle*: clonogenic survival. *Right*: LD90 values were determined from clonogenic survival curves using GraphPad Prism v8. Data are reported as the mean ± SD for at least three independent experiments. *p*-values were obtained using the two-tailed unpaired Student *t* test, adjusted for multiple tests by the Holm-Sidak method; ∗*p* < 0.05, ∗∗*p* < 0.01, ∗∗∗*p* < 0.001. ns, not significant. 6-4PP, 6-4 pyrimidine-pyrimidone photoproduct; NER, nucleotide excision repair.

We chose to further characterize the S phase NER defect in cells depleted for Dyrk1A, a dual specificity kinase that autophosphorylates on a tyrosine residue, and whose other known substrates are modified on serine and/or threonine. Dyrk1A exhibits broad functionality, being involved in multiple processes associated with neuronal development, transcriptional control, and cell proliferation (26). As was the case for siRNA-mediated knockdown of Dyrk1A in HeLa cells, CRISPR-Cas9 KO of *Dyrk1A* in primary LF-1 lung fibroblasts significantly inhibited 6-4PP removal during S phase (Fig. 2*A*), demonstrating that this effect is not cell type–specific. Moreover, quantification of unscheduled incorporation of the nucleoside analog EdU post-UV in G1/G2 HeLa cells did not reveal any effect of Dyrk1A knockdown on NER gap-filling, whereas control cells depleted for the essential NER factor XPA manifested a profound defect (Fig. 2*B*). We further found that knockdown of Dyrk1A sensitized HeLa cells to UV but did not exacerbate the UV sensitivity caused by knockdown of XPA (Fig. 2*C*). This indicates that Dyrk1A protects cells against UV-induced cell killing specifically by promoting NER during S phase.

### Dyrk1A depletion does not inhibit S phase NER by causing DNA replication stress

**Figure 3:**
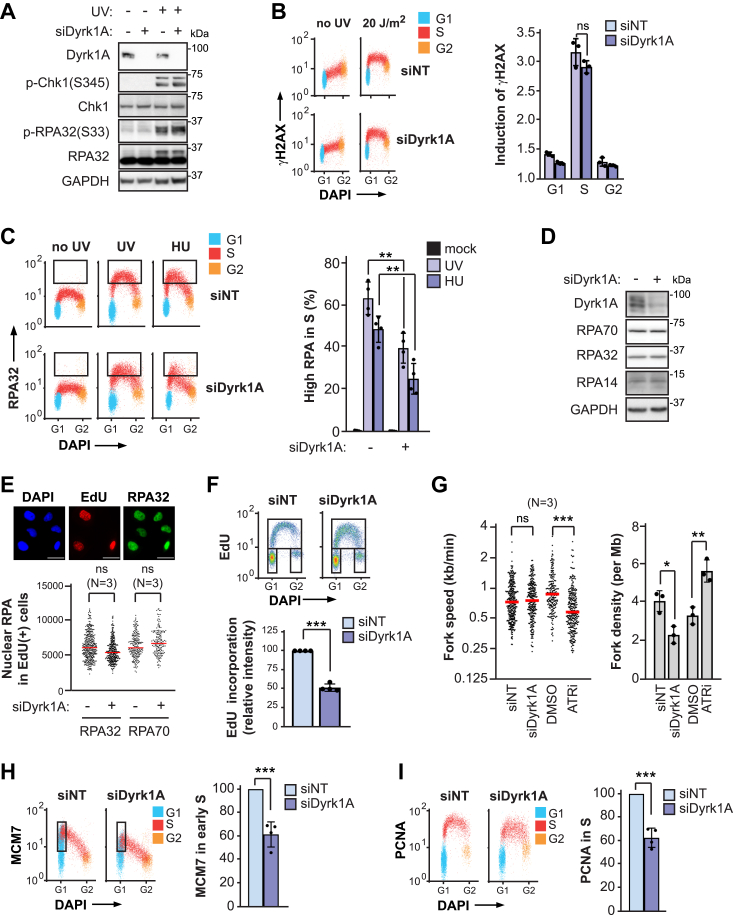
**Dyrk1A depletion does not cause replicative stress or reduced RPA availability post-UV.***A*, detection of phosphorylated Chk1 and RPA32 by immunoblotting 3 h after 20 J/m^2^ UV ± siRNA against Dyrk1A (siDyrk1A) or control (siNT). *B*, levels of γ-H2AX ± siDyrk1A were measured as a function of cell cycle 1 h after 20 J/m^2^ UV by immunofluorescence flow cytometry. *C*, *left*: RPA32-bound DNA was measured ± siDyrk1A by immunofluorescence flow cytometry 1 h after treatment with 20 J/m^2^ UV or 10 mM hydroxyurea (HU). Cells in various phases of the cell cycle were gated using EdU incorporation as an S-phase marker; each phase is represented by a different color. The boxes delineate cells with elevated RPA-bound DNA. *Right*: quantification of S phase cells with elevated RPA-bound DNA from the *left panel*. *D*, immunoblot analysis of RPA subunits from total cellular extracts treated with siDyrk1A or siNT. *E*, total nuclear levels of RPA32 and RPA70 were measured by immunofluorescence. Nuclear intensities for each subunit were quantified from microscopy images. The scale bar represents 20 μm. Average intensities of RPA32 or RPA70 in EdU+ cells were determined for siDyrk1A or siNT controls. *F*, *top*: cell cycle distribution assessed by DNA content analysis (DAPI) and EdU incorporation. *Bottom*: fluorescence intensity of EdU incorporation in S phase ± siDyrk1A. *G*, fork speed and global fork densities were measured by DNA combing in HeLa cells treated with siDyrk1A (*versus* siNT) or with the ATR inhibitor (ATRi) VE-821 (*versus* dimethyl sulfoxide (DMSO)). *H*, *left*: MCM7-bound DNA detected by flow cytometry. Cells in various phases of the cell cycle were gated using EdU incorporation as an S phase marker; each phase is represented by a different color. *Right*: fluorescence intensity of MCM7-bound DNA in early S phase (boxed in *left panel*) for siDyrk1A relative to siNT controls. *I*, same as (*H*), but for PCNA-bound DNA in cells throughout S-phase. Data are reported as the mean ± SD, except for quantification of microscopy data where medians are shown, for at least three independent experiments. *p*-values were derived using the two-tailed unpaired Student *t* test, adjusted for multiple tests by the Holm-Sidak method where applicable; ∗*p* < 0.05, ∗∗*p* < 0.01, ∗∗*∗p* < 0.001. ns, not significant. ATRi, ataxia telangiectasia and rad 3-related kinase inhibitor; RPA, replication protein A.

As mentioned earlier, S phase NER defects can be caused by excessive sequestration of RPA on ssDNA generated during periods of severe replicative stress (10, 11, 14). We therefore sought to examine the impact of Dyrk1A knockdown on the UV-induced replicative stress response. Phosphorylation of histone H2AX(S139), Chk1(S345), and RPA32(S33), well-known markers for activation of replicative stress–induced signaling, were not elevated post-UV in Dyrk1A-depleted *versus* control HeLa cells (Fig. 3, *A* and *B*). Interestingly, the level of DNA-associated RPA32, which is clearly expected to increase during replicative stress (27), was actually reduced upon Dyrk1A depletion *versus* the situation for nontargeting siRNA controls, following treatment with either UV or the replication-blocking drug hydroxyurea (Fig. 3*C*). The observed reduction in DNA-bound RPA32 after UV was not due to any effect of Dyrk1A on the total amount (Fig. 3*D*) or nuclear localization (Fig. 3*E*) of RPA subunits at the time of irradiation. Instead, Dyrk1A depletion caused a reduction in total EdU incorporation during S phase (Fig. 3*F*) and increased the duration of S phase (Fig. S2). DNA combing further showed that knockdown of Dyrk1A did not affect DNA replication fork speed but decreased the global density of active replication forks (Fig. 3*G*). In contrast, control cells treated with an ATR inhibitor exhibited significantly increased fork density, as expected (28) (Fig. 3*G*). The above data suggest that reduced RPA loading on DNA in Dyrk1A-depleted cells is likely due to a decrease in the number of active replication forks during S phase. Consistent with this notion, the abundance of DNA-associated MCM7 (a subunit of the minichromosome maintenance replicative helicase complex (MCM)), and of the DNA replication factor proliferating cell nuclear antigen (PCNA), were also significantly decreased in S phase cells lacking Dyrk1A (Fig. 3, *H* and *I*). This is in agreement with previous results indicating that Dyrk1A depletion decreases the duration of G1 (16) which, in turn, would be expected to cause a reduction in origin licensing, thereby diminishing the number of active replication forks in the subsequent S phase (29). Based on the overall data, we conclude that defective S phase NER caused by lack of Dyrk1A is not a consequence of replicative stress–induced reduction in RPA availability.

A recent study showed that EdU is a substrate for NER (30). This raises the possibility that the EdU used to label S phase cells may compete with CPD and 6-4PP for removal *via* NER, thereby potentially confounding our interpretation of the effects of Dyrk1A knockdown on DNA replication dynamics post-UV. Contrary to this notion, we found that a pulse of EdU (equal in time to that used in our experiments) prior to UV treatment did not affect the efficiency of 6-4PP removal (Fig. S1*E*), indicating that the presence of EdU does not detectably influence the excision of DNA photoproducts under our experimental conditions.

### Dyrk1A depletion causes S phase–specific NER defects through overexpression of cyclin D1

**Figure 4:**
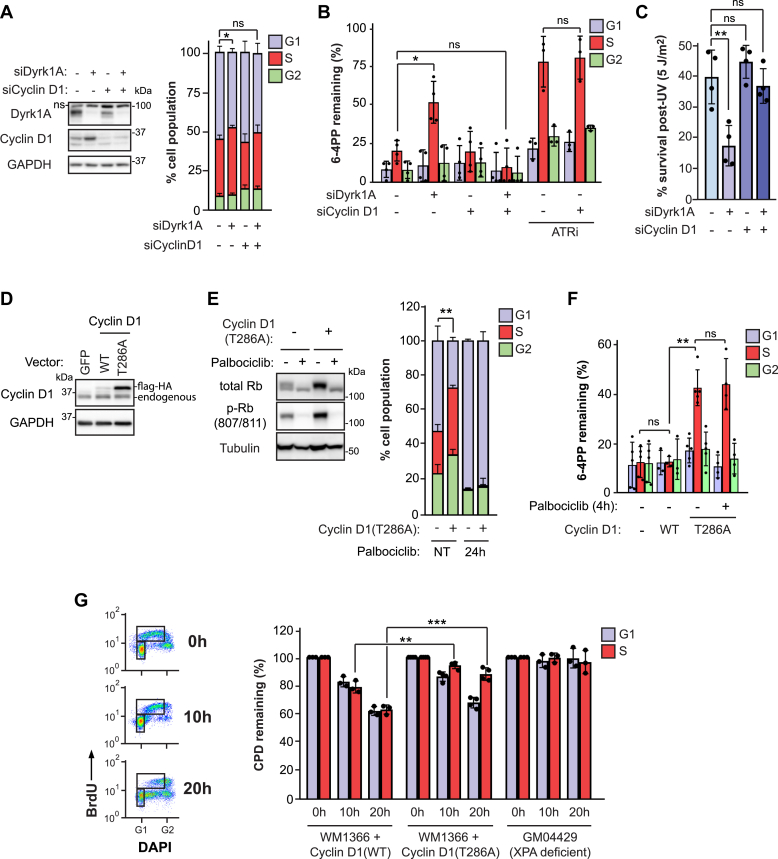
**Dyrk1A promotes NER by regulating cyclin D1 stability.***A*, *left*: immunoblot from total HeLa cell extracts, following treatment with siRNA against Dyrk1A (siDyrk1A) and/or cyclin D1 (siCyclin D1). *Right*: cell cycle distribution assessed by DNA content analysis (DAPI) and EdU as in Figure 3*F*. *B*, quantification of 6-4PP removal in HeLa cells ± siDyrk1A and/or siCyclin D1. VE-821 (10 μM) was employed as ATR inhibitor (ATRi). *C*, clonogenic survival post-UV (5 J/m^2^) in HeLa cells ± siDyrk1A and/or siCyclin D1. *D*, expression of Flag-HA-cyclin D1 (WT or T286A) in WM1366 using a retroviral construct. Flag-HA-GFP is used as a control. *E*, *left*: immunoblot of retinoblastoma protein (Rb) and phospho-Rb (p-Rb) in WM1366 ± cyclin D1(T286A), pretreated or not for 4 h with 10 μM palbociclib. *Right*: overexpression of cyclin D1 (T286A) in WM1366 decreases the % of cells in G1 in a CDK-dependent manner. Cell cycle was assessed by flow cytometry of cells labeled with EdU and DAPI, with or without pretreatment with the CDK4/6 inhibitor palbociclib for 24 h. *F*, effect of cyclin D1(T286A) overexpression on 6-4PP removal. Cells were pretreated (or not) with palbociclib for 4 h, and the drug was maintained in the medium during post-UV incubation. *G*, effect of cyclin D1 overexpression on CPD removal. Cultures were pulsed with BrdU to label cells that were in S phase at the time of irradiation. Post-UV incubations were carried out in the presence of nocodazole to prevent cell division. *Left*: BrdU(−) and BrdU(+) cells were gated to select cells remaining in G1 and S, respectively, as indicated. *Right*: % of CPD remaining in G1 and S at 10 h and 20 h post UV. The XPA-null human fibroblast line GM04429 was used as a control. Data are reported as the mean ± SD for at least three independent experiments. *p* values were obtained using the two-tailed unpaired Student *t* test, adjusted for multiple tests by the Holm-Sidak method where applicable; ∗*p* < 0.05, ∗∗*p* < 0.01, ∗∗∗*p* < 0.001. ns, not significant. 6-4PP, 6-4 pyrimidine-pyrimidone photoproduct; CDK, cyclin-dependent kinase; CPD, cyclobutane pyrimidine dimer; NER, nucleotide excision repair; Rb, retinoblastoma protein.

As noted earlier, among the known substrates of Dyrk1A is cyclin D1, which forms a complex with CDK4/6 that controls the G1-S transition (31). Moreover, Dyrk1A was shown to phosphorylate cyclin D1 on T286, which targets the latter for timely nuclear export and proteolytic degradation, thus preventing premature entry into S phase (16). Consistently, siRNA knockdown of Dyrk1A in HeLa cells increased cyclin D1 abundance (Fig. 4*A*, left panel) and caused a reduction in the proportion of cells in G1 in a cyclin D1–dependent manner (Fig. 4*A*, right panel). Strikingly, the S phase NER defect and reduced viability post-UV in cells lacking Dyrk1A were both completely rescued upon codepletion of cyclin D1 (Fig. 4, *B* and *C*). In contrast, depletion of cyclin D1 did not nonspecifically rescue defective S phase NER caused by ATR inhibition (9) (Fig. 4*B*).

To further characterize the influence of nuclear cyclin D1 overexpression on NER, we expressed either WT cyclin D1 or the nonphosphorylatable variant cyclin D1 (T286A), both epitope-tagged with Flag-hemagglutinin (Flag-HA), in the human melanoma cell line WM1366, which we previously determined to be S phase NER-proficient (32). As expected, Flag-HA-cyclin D1 (T286A) (hereafter cyclin D1 [T286A]) was expressed at higher levels than either Flag-HA-WT cyclin D1 or endogenous WT cyclin D1 (Fig. 4*D*) (16). The overexpressed cyclin D1 (T286A) variant was functional since it elevated both retinoblastoma protein (Rb) phosphorylation and the fraction of cells in S phase, while concomitantly reducing the proportion of cells in G1; moreover, these phenotypes were reversed by pharmacological inhibition of CDK4/6 using palbociclib (Fig. 4*E*). In line with our result in Dyrk1A-depleted HeLa cells, overexpression of cyclin D1 (T286A) generated an S phase NER defect in WM1366 relative to controls expressing either Flag-HA-WT-cyclin D1 or Flag-HA-GFP (Fig. 4*F*). S phase NER was also strongly inhibited in LF-1 primary lung fibroblasts overexpressing cyclin D1 (T286A) *versus* counterparts expressing Flag-HA-GFP (Fig. S3, *A*–*C*). In WM1366, pretreatment with palbociclib did not rescue the S phase NER defect caused by cyclin D1 (T286A) overexpression (Fig. 4*F*), indicating that cyclin D1 regulates S phase NER in a CDK-independent manner.

We also evaluated the effect of cyclin D1 (T286A) overexpression on the removal of CPD, which are processed by NER considerably more slowly than 6-4PP, that is, typically 50% removal after 24 h *versus* 80 to 100% removal after 5 h, respectively. Cell cycle progression resumes by ∼10 h post-UV under our experimental conditions, after which the S phase population becomes “contaminated” with cells that had been irradiated in G1. We therefore used a previously described modification of our flow cytometry assay (32), which uses BrdU to label and track cells that were in S at the time of UV treatment and that remain in S throughout the experiment (Fig. 4*G*, left). We observed that WM1366 cells expressing cyclin D1 (T286A) remove CPD significantly less efficiently during S phase at 10 and 20 h post-UV, relative to the situation for cells expressing Flag-HA-WT cyclin D1 (Fig. 4*G*, right). As expected, the control XPA-deficient cell line GM04429 exhibited profoundly deficient CPD removal in either G1 or S at both time points. We therefore conclude that cyclin D1 (T286A) overexpression inhibits NER-mediated removal of both 6-4PP and CPD during S phase.

**Figure 5:**
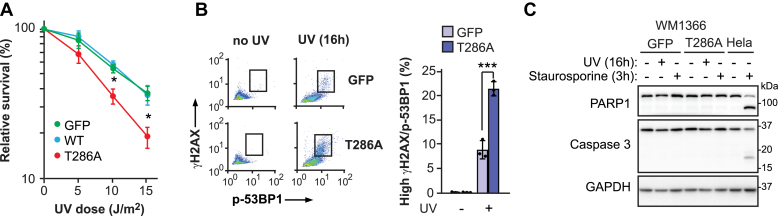
**Overexpression of cyclin D1 (T286A) sensitizes WM1366 to UV killing.***A*, UV sensitivity measured by clonogenic survival. *B*, detection of phospho-53BP1(S1778) and γ-H2AX in WM1366 cells at 16 h after irradiation with 20 J/m^2^ UV. *C*, apoptosis assessed by cleavage of caspase 3 and of poly(ADP-ribose) polymerase 1 (PARP1) at 16 h post-UV. Cells treated with 1 μM staurosporine for 3 h was used as a positive control. Data are reported as the mean ± SD for at least three independent experiments. *p* values were obtained using the two-tailed unpaired Student *t* test, adjusted for multiple tests by the Holm-Sidak method where applicable; ∗*p* < 0.05, ∗∗∗*p* < 0.001. ns, not significant.

Consistent with its negative impact on S phase NER, cyclin D1 (T286A) overexpression in WM1366 significantly increased UV-induced cytotoxicity compared with counterparts expressing either Flag-HA-WT cyclin D1 or Flag-HA-GFP (Fig. 5*A*). In addition, we observed higher levels of histone H2AX(S139) and 53BP1(S1778) phosphorylation, two well-established markers of the DNA damage response (33, 34), at 16 h post-UV in cyclin D1 (T286A) overexpressing cells *versus* control cells (Fig. 5*B*). This indicates that S phase NER-defective cells are subject to persistent DNA damage signaling, which may originate from, for example, stalled replication forks at unrepaired UV-induced DNA lesions that eventually collapse into double-strand breaks (35). Such signaling is unlikely to be caused by apoptosis-associated nucleolytic DNA fragmentation (36, 37), since (i) known markers of apoptosis, that is, caspase 3- and poly(ADP-ribose) polymerase 1-cleavage, were not detected at 16 h post-UV in WM1366 (Fig. 5*C*) and (ii) this cell line appears relatively resistant to apoptosis, as staurosporine treatment did not cause either poly(ADP-ribose) polymerase 1- or caspase 3- cleavage, in contrast to the situation for HeLa cells (Fig. 5*C*). The above data suggest that abnormal regulation of cyclin D1, leading to inhibition of NER specifically during S phase, generates lethal DNA lesions and cell death in melanoma cells post-UV.

### Cyclin D1 (T286A) overexpression causes S phase NER defects in a p21-dependent manner in melanoma cells

**Figure 6:**
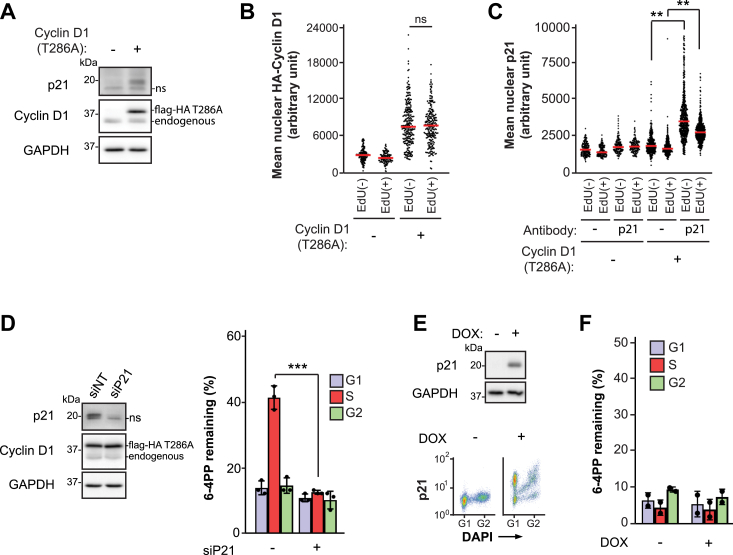
**Expression of p21 is required to sustain the S phase NER defect caused by cyclin D1 overexpression in melanoma cells**. *A*, immunoblot of p21 and cyclin D1 from total cellular extracts. *B*, nuclear levels of HA-tagged cyclin D1 in EdU(−) and EdU(+) cells measured by immunofluorescence. *C*, nuclear levels of p21 in EdU(−) and EdU(+) cells measured by immunofluorescence. *D*, removal of 6-4PP ± siRNA against p21 (siP21) in WM1366 cells overexpressing cyclin D1 (T286A). *E*, *upper*: immunoblot of p21 from WM1366 Tet-ON inducible cell line (expressing only endogenous WT cyclin D1) at 5 h after induction with doxycycline (DOX). *Lower*: cell cycle–specific analysis of p21 protein levels at 5 h after induction with DOX. *F*, removal of 6-4PP at 5 h post-UV ± DOX-induced p21 expression. *p* values comparing the medians (*B* and *C*) or means (*D*), for at least three independent experiments were obtained using the two-tailed unpaired Student *t* test, adjusted for multiple tests by the Holm-Sidak method where applicable; ∗∗*p* < 0.01, ∗∗∗*p* < 0.001. ns, not significant. 6-4PP, 6-4 pyrimidine-pyrimidone photoproduct; NER, nucleotide excision repair.

Dyrk1A depletion was previously shown to increase the levels of p21 in a cyclin D1–dependent manner (16). Consistently, we observed elevated p21 abundance upon overexpression of cyclin D1 (T286A) in WM1366 cells (Fig. 6*A*); moreover, increased nuclear expression of both cyclin D1 (T286A) and p21 was detected in S phase as well as G1/G2 cells (Fig. 6, *B* and *C*). Remarkably, siRNA-mediated depletion of p21 resulted in complete rescue of the S phase NER defect caused by cyclin D1 (T286A) overexpression (Fig. 6*D*). On the other hand, using an inducible Tet-ON system, we found that upregulation of p21 alone in WM1366 cells expressing endogenous WT cyclin D1 did not result in an S phase NER defect (Fig. 6, *E* and *F*). We conclude that elevated p21 expression is required to inhibit NER during S phase in cyclin D1 (T286A)–overexpressing melanoma cells.

## Discussion

We conducted a flow cytometry-based CRISPR/Cas9 screen to identify novel genes implicated in NER. To our knowledge, this represents the first genome-wide functional screen where the efficiency of UV damage repair is the endpoint. While genes encoding NER pathway proteins were well-represented among the top hits, we also recovered *POLH*, encoding pol eta which is known to mediate replicative bypass of UV-induced CPD (38); moreover, we previously demonstrated that lack of this translesion DNA polymerase results in abrogation of NER uniquely during S phase (8). The above considerations provide confidence in our screening strategy and show that it can identify genes which influence DNA photoproduct removal in a cell cycle–specific manner.

Remarkably, depletion of all seven genes selected for validation in the present study generated significant defects in 6-4PP removal strictly in S phase. As discussed earlier, we and others previously demonstrated that such defects can be caused by abnormal replicative stress responses (*e.g.,* in ATR- and pol eta-deficient cells), leading to excessive sequestration of heterotrimeric RPA *in trans* at persistently stalled replication forks or aberrantly activated origins of replication, which in turn prevents the essential action of this complex in NER (10, 11, 14). Moreover, our published results demonstrating that S phase NER defects are (i) recovered in a majority of MM cell lines (32) and (ii) strongly associated with chemosensitivity in ovarian cancer cells (11) highlight the biological and clinical relevance of this cell cycle–specific DNA repair phenotype. Taken together, our findings suggest that a substantial proportion of NER-modulating genes within the human genome, when mutated, cause significant defects in UV damage repair exclusively during S phase. Further investigations are warranted to evaluate whether replicative stress–associated RPA sequestration, and/or other mechanisms, underlie such defects.

We focused our efforts on characterization of the S phase NER defect caused by lack of Dyrk1A, a kinase which regulates multiple cellular pathways implicated in neurogenesis, transcription, cell cycle control, and cancer development (39). While we initially suspected that Dyrk1A knockdown might reduce S phase NER efficiency *via* deregulation of the replicative stress response leading to reduction of RPA availability, our data demonstrate that this is not the case. Rather we found that Dyrk1A regulates S phase NER through phosphorylation of cyclin D1 on T286 (16), a modification that promotes cyclin D1 nuclear export and proteolytic degradation (40, 41). Cyclin D1 forms a complex with CDK4/6 to catalyze phosphorylation of Rb, leading to E2F transcription factor activation and consequent expression of genes required for the G1-S transition (31). As such, Dyrk1A-dependent phosphorylation of cyclin D1 prevents premature entry into S phase and concomitant genomic instability/enhanced cellular proliferation (16). It should be mentioned that in addition to Dyrk1A, other cellular kinases including GSK3β, ERK1/2, and p38 have been shown to phosphorylate cyclin D1 on T286, which can also promote its cytoplasmic relocalization/proteasomal degradation (42, 43). Nonetheless, in our hands, loss of Dyrk1A alone was sufficient to stabilize and significantly upregulate cyclin D1.

Cyclin D1 is a major driver of multiple cancers (44) including MM (45), through overexpression in either the cytoplasm or nucleus where it promotes tumor invasion/metastasis or deregulation of cell cycle control/enhanced proliferation, respectively. To exert an oncogenic effect in the nucleus, cyclin D1 must be aberrantly stabilized in conjunction with inhibition of its cytoplasmic relocalization (46), as exemplified by the situation for the nonphosphorylatable cyclin D1(T286A) variant used here (40). In addition to its well-established role in cell cycle regulation, various noncanonical CDK-dependent and/or CDK-independent roles for nuclear cyclin D1 have been reported, for example, in transcription and DNA double-strand break repair (47, 48). Moreover, cyclin D1 is known to associate with the essential DNA replication factor PCNA (49, 50, 51), thereby forestalling interaction of the latter with DNA polymerases to inhibit DNA synthesis (49). One early investigation indicated that, in UV-exposed human fibroblasts, cyclin D1 overexpression inhibits PCNA-dependent NER gap-filling during G1 (52). Our data are not consistent with this, since (i) NER defects caused by cyclin D1 overexpression were observed only in S phase and (ii) quantification of UDS in HeLa cells revealed no significant impairment of NER gap-filling in either G1 or G2 post-UV upon Dyrk1A knockdown.

p21 (like cyclin D1) is known to interact with PCNA. Indeed, p21 strongly binds this DNA replication factor *via* a canonical PCNA-interacting protein box motif (53). However, while there is a general agreement that p21 (like cyclin D1) can interfere with DNA replication *via* PCNA interaction, any influence of this protein on PCNA-dependent gap-filling during NER, or any other step of this repair pathway, remains controversial. Two studies using human cell-free extracts reported that p21 exerts no effect on PCNA-dependent NER gap-filling (54, 55), although another indicated that it strongly inhibits this process (56). Yet another investigation supported this latter result *in vitro*, as well as *in vivo* (57). While our data indicate that Dyrk1A depletion and consequent cyclin D1/p21 overexpression does not negatively impact NER gap-filling during G1 or G2, the possibility of such an impact specifically in S phase cannot be ruled out. We emphasize that this possibility would be challenging to evaluate; indeed, quantification by UDS of NER gap-filling during S phase is technically unfeasible, since comparatively weak EdU incorporation signals emanating from this process are dwarfed by those resulting from chromosomal DNA replication (15). While we observed complete rescue of defective S phase NER in cyclin D1 (T286A)–overexpressing melanoma cells upon p21 depletion (without reducing levels of the former), cells expressing only endogenous WT cyclin D1 did not manifest any NER defect following inducible ectopic overexpression of p21. Our overall results thus indicate that co-overexpression of cyclin D1 and p21 is essential to generate the S phase NER defect caused by Dyrk1A depletion, although the precise mechanism remains unclear.

Overwhelming evidence demonstrates that efficient removal of UV-induced DNA photoproducts during sunlight exposure is critical for protection against skin cancer. Nonetheless, to date, few data support the plausible expectation that sporadic MM in the general population is frequently characterized by defective NER. We posit that this apparent paradox may be explained, in part, by the fact that no previous studies to our knowledge (with the exception of our own revealing a prevalence of S phase NER defects among model MM cell lines (32)) have considered the possibility that NER might be frequently inhibited in MM in a cell cycle–specific manner. As such, our data showing that aberrant accumulation of nuclear cyclin D1 generates defects in NER specifically during S phase may harbor major implications for MM development. Moreover, this may extend to other major cancers; for example, carcinoma of the lung, often characterized by the expression of oncogenic cyclin D1, is closely associated with exposure to the ubiquitous environmental carcinogen benzo(a)pyrene that generates highly mutagenic helix-destabilizing DNA adducts repaired exclusively by NER.

## Experimental procedures

### Cell culture

LF-1 primary human lung fibroblasts (18), a gift from John Sedivy (Brown University) and GM04429 XPA-deficient skin fibroblasts (Coriell Institute) were grown in Eagle's minimal essential medium containing 15% fetal bovine serum, essential and nonessential amino acids, vitamins, and antibiotics (Life Technologies). HeLa cells (ATCC) were cultured in Dulbecco's modified Eagle's medium + 10% fetal bovine serum and antibiotics. WM1366 melanoma cells (Coriell Institute) were cultured as described (32). All cell lines were authenticated by short tandem repeat analysis (McGill University Genome Center) and routinely tested for *mycoplasma* contamination by staining with DAPI.

### Reagents and plasmids

Chemical inhibitors, antibodies, siRNAs, and DNA oligonucleotides used in this study are listed in Supporting Experimental Procedures. Plasmids and siRNAs were transfected using Lipofectamine 2000 and RNAiMax, respectively (Life Technologies). LentiCRISPRv2 (19) was a gift from Feng Zhang (Addgene plasmid # 52961). Complementary DNAs (cDNA) encoding either cyclin D1 WT or cyclin D1 (T286A) were PCR amplified from pcDNA cyclin D1 HA and pcDNA cyclin D1 HA T286A (Addgene plasmids #11181 and #11182, respectively; gifts from Bruce Zetter (58)) and cloned into pDONR221 using Gateway BP Clonase (Life Technologies). Entry clones were recombined with MSCV-N-Flag-HA-IRES-PURO (59) (a gift from Wade Harper, Addgene plasmid #41033) using Gateway LR Clonase II (Life Technologies). The coding sequence of p21 was PCR amplified from flag-p21-WT (60) (a gift from Mien-Chie Hung, Abcam #16240) and cloned into pRetro-X-tight-PUR (Clontech), using NotI and EcoRI sites. To generate WM1366-TetON-p21, WM1366 was transduced with pLenti-CMV-rtTA3-Blast (a gift from Eric Campeau, Addgene #26429) and selected with 20 μg/ml blasticidin (Life Technologies). The cell line was then transduced with pRetro-X-tight-PUR-p21 and selected with 1 μg/ml puromycin (Life Technologies). Induction of p21 was carried out with 5 μg/ml of doxycycline (Bioshop Canada) for indicated times.

### Cell irradiation

Cell monolayers were washed with PBS and covered with a thin layer of PBS, followed by irradiation with monochromatic 254-nm UV (hereafter UV) using a G25T8 germicidal lamp (Philips). The fluence was 0.7 J/m^2^/s, as measured with a Spectroline DRC 100× digital radiometer equipped with a DIX-254 sensor (Spectronics Corporation).

### Flow cytometry–based NER assay

Repair of 6-4PP was evaluated as a function of cell cycle as described (9). Briefly, replicate exponentially growing cultures were irradiated with 20 J/m^2^ of UV (or mock-irradiated) and harvested either immediately (0 h time point) or following 5 h incubation to allow repair. Cells were then fixed, permeabilized, and double-stained with DAPI and Alexa647-conjugated anti-6-4PP antibody. Bivariate flow cytometry analysis was used to quantify 6-4PP removal for populations gated in each phase of the cell cycle. Data were acquired using an LSR II flow cytometer (BD Biosciences) and analyzed with FlowJo software v10 (https://www.bdbiosciences.com/en-ca/products/software/flowjo-v10-software).

Removal of CPDs as a function of cell cycle was measured essentially as described (32). Briefly, cells were irradiated with 15 J/m^2^ of UV, followed by incubation with 30 μM BrdU (Sigma-Aldrich) for 2 h to label cells in S phase at the time of irradiation. Cells were then washed with PBS, and fresh medium containing 200 ng/ml nocodazole to prevent cell division was added. Samples were collected at 10 h and 20 h post-UV. The 0 h time point and mock-UV control were labeled with BrdU for 1 h prior to UV irradiation and harvested immediately. Cells were triple stained using Alexa488-conjugated anti-CPD, Alexa647-conjugated anti-BrdU, and DAPI. BrdU and DAPI bivariate plots were used to gate BrdU(−) and BrdU(+) cells remaining in G1 and S, respectively, at each time point. Data were acquired and analyzed as outlined above for 6-4PP.

### CRISPR/Cas9 screen

The GeCKO v2 human CRISPR/Cas9 pooled library (a gift from Feng Zhang, Addgene #1000000048) was used as described (19) to infect a population of LF-1 primary fibroblasts. Following puromycin selection, at day 10, 10^8^ sgRNA-transduced cells were irradiated with 20 J/m^2^ UV and labeled with DAPI and anti-6-4PP antibody at 5 h post-UV, and NER-deficient cells were sorted using flow cytometry. Genomic DNA was isolated, and a sample was stained with PicoGreen (Life Technologies), followed by quantification using a TBS-380 fluorometer (Turner Biosystems). PCR of genomic DNA and next generation sequencing were carried out as described (19) to identify sgRNAs from control *versus* NER-deficient populations (see Supporting Experimental Procedures for details). Sequencing adapters were removed using Cutadapt software (https://cutadapt.readthedocs.io/en/stable/) (61). MAGeCK software version 0.5.6 (https://sourceforge.net/projects/mageck/) (20) was used to generate sgRNA read count data, enriched guides, and gene-level rankings. GO term enrichment was determined with the preranked tool from the GSEA desktop app version 4.1.0 and the Molecular Signatures Database version 7.2 (https://www.gsea-msigdb.org/gsea/index.jsp) (62, 63), using the -log10(*p*-value) of the top 150 genes obtained by MAGeCK analysis.

### Clonogenic survival

Clonogenic survival post-UV was evaluated as described (32). LD90 values for individual survival curves were determined using GraphPad Prism v8 (https://www.graphpad.com/).

### Immunoblotting

Immunoblotting was performed on whole-cell extracts using standard protocols. Antibodies are listed in Supporting Experimental Procedures. Imaging was performed using ECL prime reagent (GE healthcare) with an Azure c600 instrument (Azure Biosystems).

### Cell cycle analysis

Cells were labeled with EdU and DAPI and analyzed by flow cytometry as described (64). Data were acquired using an LSR II flow cytometer (BD Biosciences) and analyzed with FlowJo software v10.

### Detection of proteins by flow cytometry

DNA-bound RPA subunits were detected by flow cytometry as described (64). In the case of PCNA and MCM7, the same protocol was used with the following modifications: after fixation, cells were resuspended in 0.5 ml PBSB (PBS + 0.1% bovine serum albumin), mixed with 3 ml of −20 °C methanol, and incubated at −20 °C for 15 min. Cells were pelleted at 500*g* for 2 min at 4 °C, washed with 2 ml of PBSB, and washed again with 0.5 ml of 1 X BD Perm/Wash buffer (BD Biosciences) before antibody labeling. Detection of p21 was carried out as above except cells were fixed with 4% formaldehyde in PBS and then permeabilized in cold PBS containing 0.1% Triton X-100 and 0.3 M sucrose on ice for 10 min. In all cases, data were acquired using an LSR II flow cytometer (BD Biosciences) and analyzed using FlowJo software v10.

### Immunofluorescence microscopy

For detection of total RPA32, RPA70, and HA-cyclin D1, cells were pulsed with 10 μM EdU for 20 min, washed with PBS, and fixed with 4% formaldehyde in PBS for 30 min at room temperature. Cells were then permeabilized in 0.1% Triton X-100 in PBS for 10 min, followed by blocking in PBS containing 3% bovine serum albumin overnight at 4 °C. Primary antibodies were added for 3 h at room temperature. EdU was then conjugated to Alexa 647 using click-iT chemistry as described (64), followed by DAPI staining. Detection of p21 was performed as above except that 0.2% Triton X-100 was used and blocking was at room temperature in PBS + 3% goat serum + 0.05% Tween-20 for 1 h. Primary antibody was added overnight at 4 °C. Images were acquired with a DeltaVision Elite system (GE Healthcare). Nuclear fluorescence signals were quantified using custom software as described (10).

### DNA combing

DNA combing was performed as described (65, 66). Cells were labeled for 30 min with 30 μM CldU, followed by 30 min with 250 μM IdU. (ATRi-treated cultures were pretreated for 30 min with 10 μM VE-821 before labeling.) Genomic DNA was combed on silanized glass slides at 2 kb/μm using a FiberComb molecular combing system (Genomic Vision). DNA was denatured with NaOH, stained with anti-BrdU antibodies recognizing CldU and IdU, and counter-stained for ssDNA (see Supporting Experimental Procedures). Images were acquired with a Zeiss Axio Imager Z2 microscope or DeltaVision Elite system and analyzed using ImageJ software (version 1.52h) (https://imagej.nih.gov/ij/download.html). Fork speed (kb/min) and fork densities (per Mb) were evaluated as described (66, 67). Fork densities were normalized for the % of cells in S phase, as determined by flow cytometry.

### Unscheduled DNA synthesis

NER gap-filling was evaluated by UDS assay using fluorescence microscopy as described (68). Briefly, cells were irradiated with 20 J/m^2^ UV (or mock irradiated) and then incubated for 2 h in Dulbecco's modified Eagle's medium without serum, containing 10 μM EdU and 1 μM 5-fluoro-2′-deoxyuridine (Sigma-Aldrich). Images were acquired with a DeltaVision Elite system and nuclear fluorescence signals quantified using custom software as described (10). The relative UDS (%) for each sample was calculated as the median intensity of nuclear EdU divided by that of the UV-treated siNT control. Cells in S phase, that is, with saturated EdU signal, were excluded from the analysis.

### Statistical analysis

All experiments were performed independently at least three times (biological replicates). Data are reported as the mean ± SD, except for quantification of microscopy data where medians are shown. Significance was determined with the two-tailed unpaired student *t* test and adjusted for multiple tests (Holm-Sidak method) where applicable. Statistical analyses were performed using GraphPad Prism v8; *p*-values are as follows: (∗) *p*< 0.05, (∗∗) *p*< 0.01, (∗∗∗) *p*< 0.001, and (ns) *p*-value not significant.

## Data availability

Data is available from the corresponding author upon request.

## Supporting information

This article contains supporting information.

## Conflict of interest

The authors declare that they have no conflicts of interest with the contents of this article.

## Supporting information

Supportng informaion
