# Supplementary material for: A genome-wide screen reveals that Dyrk1A kinase promotes nucleotide excision repair by preventing aberrant overexpression of cyclin D1 and p21
Source: J Biol Chem. 2023 Jun 9;299(7):104900. doi: 10.1016/j.jbc.2023.104900 (PMC10339196; doi:10.1016/j.jbc.2023.104900)
Supplement: Supportng informaion [file mmc1.pdf]

SUPPORTING INFORMATION

A genome-wide screen reveals that Dyrk1A kinase promotes nucleotide excision repair by preventing aberrant overexpression of cyclin D1 and p21

François Bélanger[1], Cassandra Roussel[1], Christina Sawchyn[1,2], Edlie St-Hilaire[1], Sari Gezzar-Dandashi[1,3], Aimé Boris Kimenyi Ishimwe[1,3], Frédérick Antoine Mallette[1,2,3,4], Hugo Wurtele[1,3,4*] and Elliot Drobetsky[1,3,4 *]

1. Centre de Recherche de l'Hôpital Maisonneuve-Rosemont, 5415 boulevard de l'Assomption, Montréal, Québec, Canada H1T 2M4

2. Department of Biochemistry and Molecular Medicine, Université de Montréal, 2900 Édouard-Montpetit, Montréal, Québec, Canada, H3T 1J4

3. Molecular Biology Program, Université de Montréal, 2900 Édouard-Montpetit, Montréal, Québec, Canada, H3T 1J4

4. Department of Medicine, Université de Montréal, 2900 Édouard-Montpetit, Montréal, Québec, Canada, H3T 1J4

Contains:

- Figure S1
- Figure S2
- Figure S3
- Table S1 (separate xlsx file)
- Table S2 (separate xlsx file)
- Table S3
- Supporting experimental procedures

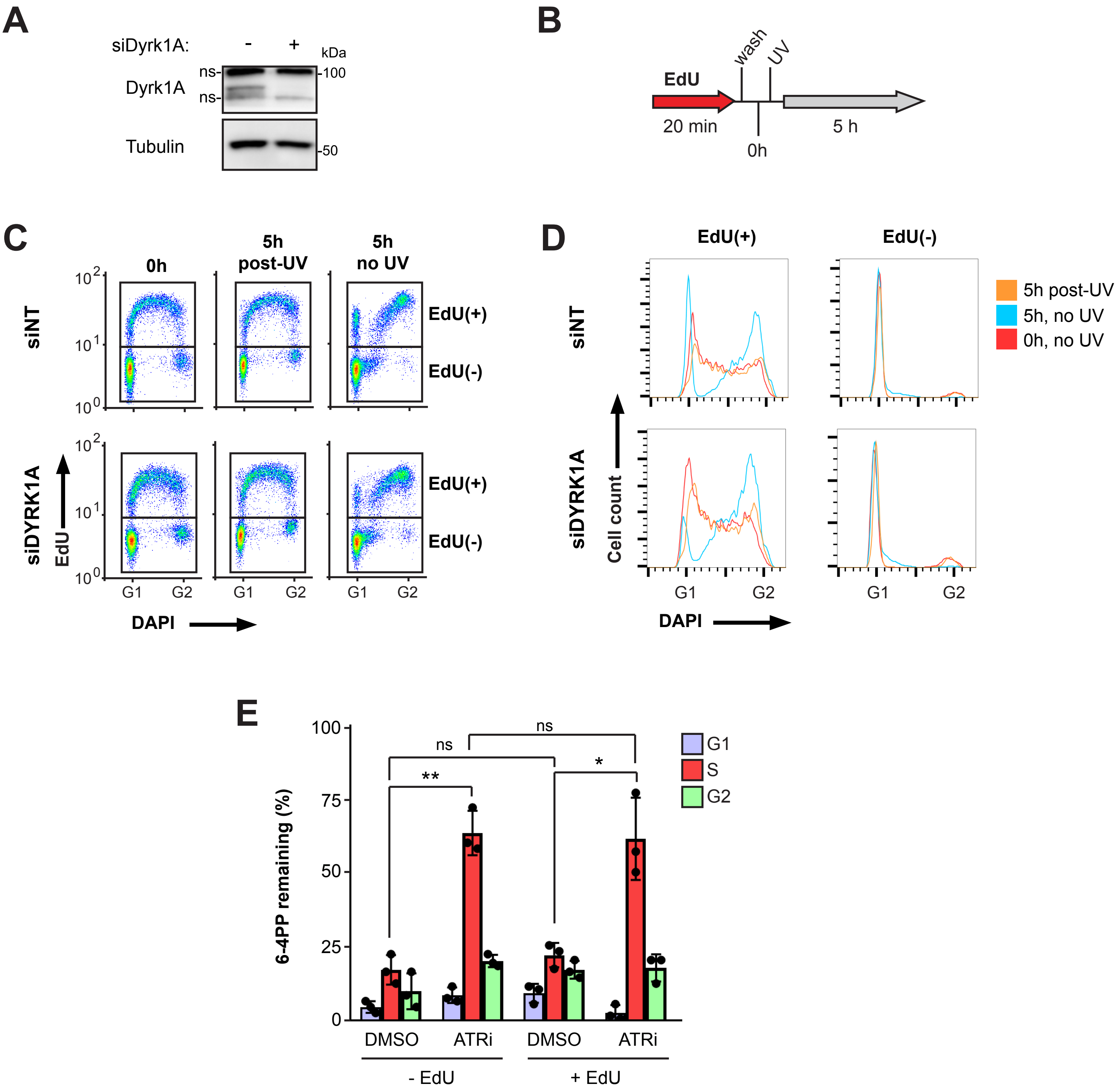

Figure S1.

Progression of HeLa cells through the cell cycle post UV.

A) Western blot showing Dyrk1A levels at 48h post-transfection with siDyrk1A or non-targeting control. **B)** Cells were pulsed with EdU for 20 min and washed with PBS. A sample was collected immediately after the EdU pulse (0h). Samples were irradiated with UV (or mock-irradiated) and harvested 5h later. **C)** Flow cytometry of cells labeled with DAPI and EdU. Boxes show gating of EdU(-) cells, and of EdU(+) cells which were in S at the time of UV irradiation. **D)** Cell cycle distribution of (DAPI-stained) EdU(-) and EdU(+) populations as gated in panel C. In the absence of UV (blue line), EdU(+) cells progressed toward G2/M, with a fraction cycling back into G1. A fraction of EdU(-) cells also entered into S. At 5h post-UV (orange line), there was almost no progression of EdU(+) cells (compare with 0h control; red line). There were also no EdU(-) cells entering into S. **E)** Hela cells were pulsed or not for 20 min with EdU as in panel B, and the efficiency of 6-4PP excision was measured as in Figure 1C. As a control, ATR inhibitor is used to produce an S-phase NER defect. P values compare the % 6-4PP remaining in S (normalized for multiple t-tests); * $p < 0.05$; ** $p < 0.01$.

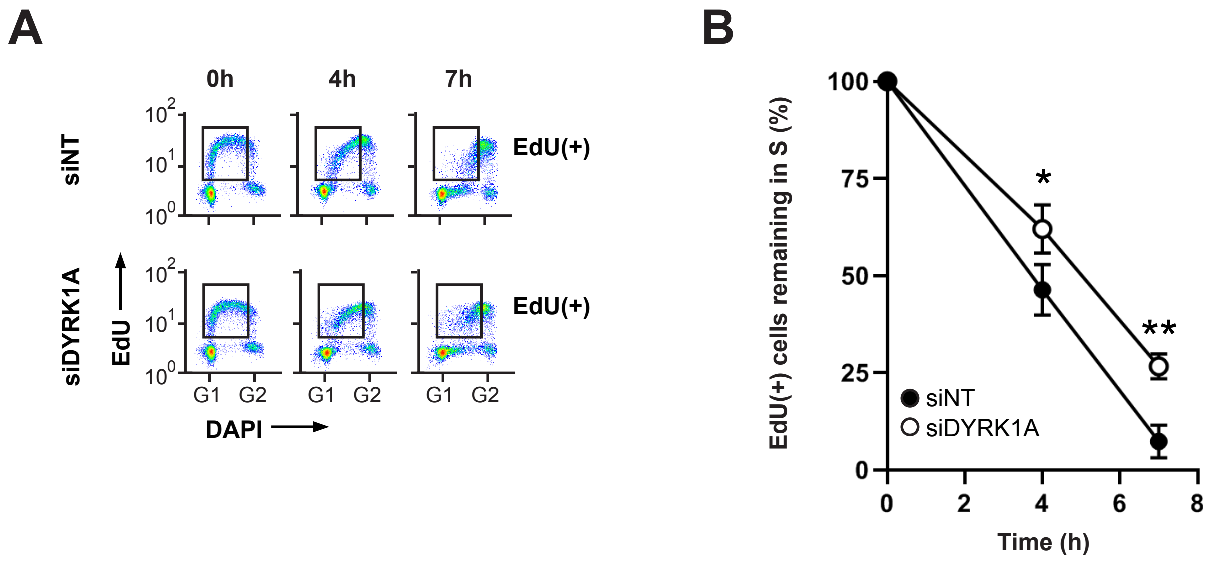

Figure S2

Dyrk1A depletion increases the duration of S phase in the absence of DNA damage.

A) Cells were pulsed with EdU, washed and incubated without EdU for indicated times in the presence of nocodazole. The black boxes represent the position of the EdU(+) cells still in S. **B)** % of EdU(+) cells remaining in S at different times relative to 0h. Values are average +/- SD of 3 independent experiments (P values are normalized for multiple t-tests: * $p < 0.05$; ** $p < 0.01$).

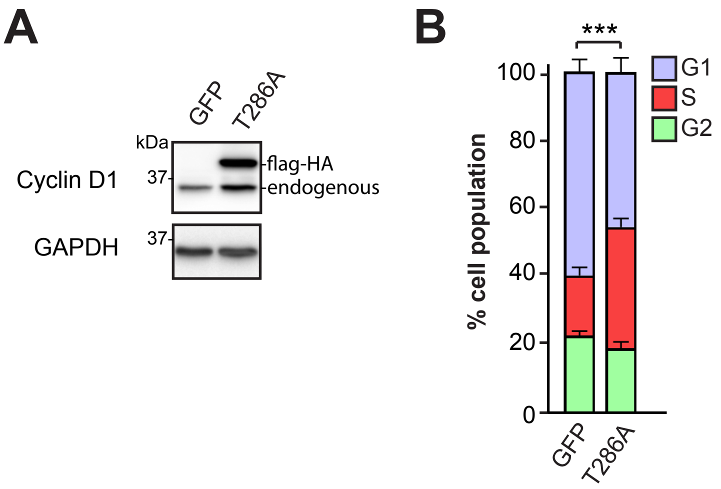

Figure S3.
Stable overexpression of cyclin D1 (T286A) inhibits NER during S phase in LF-1 primary human fibroblasts.
A) Western blot of cyclin D1 using total protein extracts. **B)** Proportion of cells in each phase of the cell cycle. **C)** Removal of 6-4PP at 5h post UV. Values are average +/- SD of 3 independent experiments (unpaired t test: ** $p < 0.01$; ***$p < 0.001$).

Table S3
RT-qPCR quantification of siRNA knock-down efficiencies in Hela cells

mRNA	% remaining	Primer pairs used for qPCR	
HPRT	control	FWD	CCCTGGCGTCGTGATTAGTG
		REV	TCGAGCAAGACGTTCAGTCC
EHD4	10 +/- 2	FWD	CGCCGTGATGTATGGAGAGA
		REV	GGGAGCTGTGAGCACATGAA
SPATC1L	4 +/- 2	FWD	CCAGGAGGAGCTACTACCTCAA
		REV	CCACCGTGAAGCCGTAGAG
ZNF182	23 +/- 1	FWD	GGCAGCAGGTTACCAAACCA
		REV	GGTCATCCTGATGTTGCCTCT
C6ORF62	14 +/- 4	FWD	TCTCTAGCTGACCAGTTTGACT
		REV	TCTCGCACACCTTTCAGGAT
DYRK1A	25 +/- 3	FWD	TTGTCATGTTACAGAGGCGG
		REV	CCCTGTTGGTGTCTTCGCTT
ZC3HC1	7 +/- 5	FWD	ACGCGAAGGACACGTCTG
		REV	AGCTCAAAGGGCTTACCTGC
POLH	40 +/- 5	FWD	AACCCAGCATTTTCGGCAAC
		REV	TCCATGTCCACGAGAGCAAC
CDA	4 +/- 3	FWD	CAGTCACTTTCCTGTGGGGG
		REV	GAGACGGCCTTCTGGATAGC
XPA	17 +/- 4	FWD	GAAGCAAAGGAAGTCCGACAG
		REV	ACACGCTGCTTCTTACTGCT

RNA was extracted with Trizol reagent (Life Technologies) and cDNA prepared with M-MuLV Reverse Transcriptase (New England Biolabs, Ipswich, MA, USA). Quantitative PCR was performed using Luna Universal qPCR Master Mix (New England Biolabs) on an ABI 7500 instrument (Applied Biosystems; Waltham, Mass, USA). Remaining mRNA (relative to siNT) was calculated by ΔΔCt using HPRT as the housekeeping control. Values are average +/- SD from at least 3 independent experiments

SUPPORTING EXPERIMENTAL PROCEDURES

Chemical inhibitors used in this study:

Name:	Catalogue #	Supplier:
Palbociclib	PZ0383	Sigma-Aldrich (Oakville, Canada)
Staurosporine	81590	Cayman Chemical Co. (Ann Arbor, MI, USA)
VE-821 ATR inhibitor	A2521	ApexBio (Houston, TX, USA)
Hydroxyurea	HYD023	Bioshop Canada (Burlington, Canada)
Nocodazole	ab120630	Abcam (Cambridge, UK)

PCR for Illumina sequencing of Gecko library:

Genomic DNA was extracted from cell pellets using SNET buffer (20 mM Tris-HCl pH8, 400 mM NaCl, 1% SDS, 5 mM EDTA) with 0.4 mg/ml proteinase K (Life Technologies, Carlsbad, CA, USA) at 55 ºC overnight, followed by extraction with phenol/chloroform (Life Technologies) and treatment with RNase A (BioBasic, Markham, Canada) for 30 minutes at 37 ºC. DNA was quantified with PicoGreen (Life Technologies) using a TBS-380 fluorimeter (Turner Biosystems, Sunnyvale, CA, USA). PCR for Illumina sequencing was performed using NEBNext High-Fidelity 2X PCR Master Mix, following manufacturer's instructions (New England Biolabs, Ipswich, MA, USA). A first PCR of 20 cycles was prepared in multiple reactions for the total amount of gDNA, at 4 µg DNA per reaction.

PCR 1 Forward: AATGGACTATCATATGCTTACCGTAACTTGAAAGTATTTCG

PCR 1 Reverse: TCTACTATTCTTTCCCCTGCACTGTTGTGGGCGATGTGCGCTCTG

Identical reactions were combined and a second PCR of 24 cycles was carried out using 5 µL of PCR1 as template with primers containing Illumina sequences and barcodes (8 identical reactions were done for each sample and combined). PCR2 products were purified on a 1.5% agarose TAE gel, visualized with SYBR-safe (Life Technologies). Sequencing was performed on an Illumina NextSeq system at the Institut de Recherche en Immunologie et Cancérologie (Montréal, Canada).

Primers used for PCR 2:

PCR 2 Forward primers (10 staggered oligos used as a mix):

V2-F0:
AATGATACGGCGACCACCGAGATCTACACTCTTTCCCTACACGACGCTCTTCCGATCTTC
TTGTGGAAAGGACGAAACACCG

V2-F1
AATGATACGGCGACCACCGAGATCTACACTCTTTCCCTACACGACGCTCTTCCGATCTTT
CTTGTGGAAAGGACGAAACACCG

V2-F2
AATGATACGGCGACCACCGAGATCTACACTCTTTCCCTACACGACGCTCTTCCGATCTAT
TCTTGTGGAAAGGACGAAACACCG

V2-F3
AATGATACGGCGACCACCGAGATCTACACTCTTTCCCTACACGACGCTCTTCCGATCTGA
TTCTTGTGGAAAGGACGAAACACCG

V2-F4
AATGATACGGCGACCACCGAGATCTACACTCTTTCCCTACACGACGCTCTTCCGATCTCG
ATTCTTGTGGAAAGGACGAAACACCG

V2-F5
AATGATACGGCGACCACCGAGATCTACACTCTTTCCCTACACGACGCTCTTCCGATCTGC
GATTCTTGTGGAAAGGACGAAACACCG

V2-F6
AATGATACGGCGACCACCGAGATCTACACTCTTTCCCTACACGACGCTCTTCCGATCTAG
CGATTCTTGTGGAAAGGACGAAACACCG

V2-F7
AATGATACGGCGACCACCGAGATCTACACTCTTTCCCTACACGACGCTCTTCCGATCTGA
GCGATTCTTGTGGAAAGGACGAAACACCG

V2-F8
AATGATACGGCGACCACCGAGATCTACACTCTTTCCCTACACGACGCTCTTCCGATCTCG
AGCGATTCTTGTGGAAAGGACGAAACACCG

V2-F9
AATGATACGGCGACCACCGAGATCTACACTCTTTCCCTACACGACGCTCTTCCGATCTAC
GAGCGATTCTTGTGGAAAGGACGAAACACCG

PCR2 Reverse primer with Illumina Index #6:

CAAGCAGAAGACGGCATACGAGATGCCAATGTGACTGGAGTTCAGACGTGTGCTCTTCC
GATCTTTCTACTATTCTTTCCCTGCACTGT

PCR2 Reverse primer with Illumina Index #12:

CAAGCAGAAGACGGCATACGAGATCTTGTAGTGACTGGAGTTCAGACGTGTGCTCTTCC
GATCTTATCTACTATTCTTTCCCCTGCACTGT

PCR primers for Gateway cloning

CCND1 Forward:

GGGGACAAGTTTGTACAAAAAAGCAGGCTTCATGGAACACCAGCTCCTGTGCTGC

CCND1 Reverse:

GGGGACCACTTTGTACAAGAAAGCTGGGTCTCAGATGTCCACGTCCCGCACGTC

EGFP Forward:

GGGGACAAGTTTGTACAAAAAAGCAGGCTTCATGGTGAGCAAGGGCGAGGAGCTG

EGFP Reverse:

GGGGACCACTTTGTACAAGAAAGCTGGGTCTTACTTGTACAGCTCGTCCATGCCG

PCR primers for p21 cloning

p21 Forward

GGAGCCGCGGCCGCCGCCACCATGTCAGAACCGGCTGGGGATG

p21 Reverse

GAGTCCGAATTCTTAGGGCTTCCTCTTGGAGAAGATC

DNA cassettes for cloning into pLentiCRISPRv2

DYRK1A #1 Forward

CACCGTATGACTAGAATCGTCTCCC

DYRK1A #1 Reverse

AAACGGGAGACGATTCTAGTCATAC

DYRK1A #2 Forward

CACCGAAGAAGCGAAGACACCAACA

DYRK1A #2 Reverse

AAACTGTTGGTGTCTTCGCTTCTTC

AAVS1 Forward

CACCGGGGGCCACTAGGGACAGGAT

AAVS1 Reverse

AAACATCCTGTCCCTAGTGGCCCCC

Antibodies used in this study

Dilutions of antibodies were 1:1000 for Western blotting (WB), 1:100 for flow cytometry (FC) and 1:300 for immunofluorescence (IF).

Name:	Catalogue #:	Supplier:	Applications:
rat anti-Tubulin	ab6161	Abcam	WB
rabbit anti-RPA1	ab79398	Abcam	IF, WB
mouse anti-RPA3	ab167593	Abcam	WB
rabbit anti-Chk1 phospho S345	2348	New England Biolabs	WB
rabbit anti-PARP	9532S	New England Biolabs	WB
mouse anti-Rb	9309	New England Biolabs	WB
rabbit anti-Rb (pS807/811)	9308	New England Biolabs	WB
mouse anti-Chk1	sc-8408	Santa Cruz (Dallas TX, USA)	WB
mouse anti-GAPDH	sc-365062	Santa Cruz	WB
mouse anti-caspase 3	sc-56053	Santa Cruz	WB
mouse anti-PCNA	sc-56	Santa Cruz	FC
rabbit anti-p21	sc-397	Santa Cruz	WB
mouse anti-p21	556430	BD (Franklin Lakes NJ, USA)	IF, FC
rabbit anti-cyclin D1	GTX108624	GeneTex (Irvine CA, USA)	WB
mouse anti-XPA	GTX72316	GeneTex	WB
rabbit anti-Dyrk1A	6255	ProSci (Fort Collins CO, USA)	WB
mouse anti-RPA2	NA18	Sigma-Aldrich	WB, IF, FC
rabbit anti-RPA2 (p-S33)	A300-246A	Bethyl (Montgomery TX, USA)	WB

mouse anti-γH2AX	JBW301	Sigma-Aldrich	FC
rabbit anti-53BP1(p-S1778)	2675	Cell Signaling (Danvers MA, USA)	FC
mouse anti-MCM7	sc-9966	Santa-Cruz	FC
mouse anti-HA (12CA5)	ab1424	Abcam	IF
mouse anti-(6-4) photoproducts	MC-082	Kamiya (Seattle, WA, USA)	FC
mouse anti-thymidine dimer	MC-062	Kamiya	FC
rat anti-BrdU	ab6326	Abcam	IF, FC
mouse anti-BrdU	347580	BD Biosciences	IF
mouse anti-ssDNA	MAB3034	Sigma-Aldrich	IF
anti-rat IgG AlexaFluor647	A21247	Life Technologies	FC
anti-rat IgG AlexaFluor594	A11007	Life Technologies	IF
anti-mouse IgG AlexaFluor647	A-21235	Life Technologies	IF
anti-mouse IgG AlexaFluor488	A11029	Life Technologies	IF, FC
anti-rabbit IgG Alexa Fluor594	A11012	Life Technologies	IF, FC
anti-rabbit IgG Alexa Fluor488	A11008	Life Technologies	IF, FC
anti-rabbit IgG-HRP	sc-2357	Santa Cruz	WB
m-IgGk-BP-HRP	sc-516102	Santa Cruz	WB
anti-Rat IgG-HRP	ab97057	Abcam	WB

siRNA oligo duplexes

Individual duplexes were purchased from Sigma-Aldrich and used as a pooled mix for each gene.

Gene	Catalogue #	Sense sequence:
DYRK1A	SASI_Hs01_00123259	GAGCUAUGGACGUUAAUUU[dT][dT]
DYRK1A	SASI_Hs01_00123260	GUGCAAUCAAGAUAGUUGA[dT][dT]
DYRK1A	SASI_Hs01_00123261	GGGUAUUCCACCUGCUCAU[dT][dT]
DYRK1A	SASI_Hs01_00123262	CUCUUUGAACCUAACACGA[dT][dT]
CCND1	SASI_Hs01_00213908	GCAUGUUCGUGGCCUCUAA[dT][dT]
CCND1	SASI_Hs01_00213909	CCACAGAUGUGAAGUUCAU[dT][dT]
XPA	SASI_Hs02_00302440	GGAAAGAAUUUAUGGAUUC[dT][dT]
XPA	SASI_Hs01_00233870	CUACUGGAGGCAUGGCUAA[dT][dT]
XPA	SASI_Hs02_00302441	GGAUUCUUAUCUUAUGAAC[dT][dT]
XPA	SASI_Hs01_00233868	CACUUUGAUUUGCCAACUU[dT][dT]
CDA	SASI_Hs01_00229075	CUGGAGAACUUCAUAAAGA[dT][dT]
CDA	SASI_Hs02_00332546	CCAGUGACAUGCAAGAUGA[dT][dT]
CDA	SASI_Hs01_00229077	GCACCAACUGGCCCGUGUA[dT][dT]
CDA	SASI_Hs01_00229078	CCUACAGGGACUGGGCAAA[dT][dT]
EHD4	SASI_Hs01_00139749	CAUCUCAGAUGAAUUCUCA[dT][dT]
EHD4	SASI_Hs01_00139750	CCUUCAUCGCCGUGAUGUA[dT][dT]
EHD4	SASI_Hs01_00139751	GCUAUAUUUAGAGUGGCAA[dT][dT]
EHD4	SASI_Hs01_00139752	GGUACUGCGCGUCUACAUU[dT][dT]
POLH	SASI_Hs01_00088494	CAUUGAUGAGGCUUACGUA[dT][dT]
POLH	SASI_Hs01_00088495	CAUAGAGAGGGAGACUGGU[dT][dT]

POLH	SASI_Hs01_00088497	CCAAAUGCCCAUUCGCAAA[dT][dT]
POLH	SASI_Hs02_00341708	GAAUAAACCUUGUGCAGUU[dT][dT]
SPATC1L	SASI_Hs01_00105335	GAGCAGACCUCCACCAAGU[dT][dT]
SPATC1L	SASI_Hs01_00105337	GCGAGUUCCUCAUCAACAC[dT][dT]
SPATC1L	SASI_Hs01_00105338	GGAGCUACUACCUCAAUGA[dT][dT]
SPATC1L	SASI_Hs01_00105339	CUCAUCAACACCUACGGAA[dT][dT]
ZC3HC1	SASI_Hs01_00207894	CAGAUUGAAUCGUCCAUGA[dT][dT]
ZC3HC1	SASI_Hs01_00207895	GGAACAACCUUCAUUGGAA[dT][dT]
ZC3HC1	SASI_Hs01_00207896	CAAGAUCAGUCUUCUCCUA[dT][dT]
ZC3HC1	SASI_Hs01_00207897	CCUUCAUUGGAAUCUACAA[dT][dT]
ZNF182	SASI_Hs01_00046638	GCAACAUCAGGAUGACCAA[dT][dT]
ZNF182	SASI_Hs01_00046639	GUCAAACCUUGGUGUACAU[dT][dT]
ZNF182	SASI_Hs01_00046640	CACUGAAAGAAAGUUGUGU[dT][dT]
ZNF182	SASI_Hs01_00046641	CUAAUCAGCAGUAUCUAUU[dT][dT]
C6orf62	SASI_Hs01_00099792	GUACUCAGGAGAUGGAUUU[dT][dT]
C6orf62	SASI_Hs01_00099794	GACAAAUAAUUAUGAAGAA[dT][dT]
C6orf62	SASI_Hs01_00099795	GUUAUACCAGUCAUGACAA[dT][dT]
C6orf62	SASI_Hs01_00099796	GAGGUUAUACCAGUCAUGA[dT][dT]
P21	SASI_Hs01_00025255	CUAAGAGUGCUGGGCAUUU[dT][dT]
P21	SASI_Hs01_00025256	CUGUCACAGGCGGUUAUGA[dT][dT]
P21	SASI_Hs01_00025257	CCCUAAUCCGCCCACAGGA[dT][dT]
